# Status Quo and Influencing Factors of Discharge Readiness of Patients with Bilateral Ureteral Stoma After Radical Cystectomy

**DOI:** 10.3389/fsurg.2022.860162

**Published:** 2022-05-25

**Authors:** Li Huang, Shuang Peng

**Affiliations:** Clinical Nursing Teaching and Research Section, The Second XiangYa Hospital of Central South University, Changsha, China

**Keywords:** bilateral ureterostomy, discharge readiness, radical cystectomy, influencing factors, status quo

## Abstract

Bladder cancer is a common malignancy of the urinary system, which occurs mostly in elderly men, and the incidence is increasing year by year. To analyze the status quo and related factors of discharge readiness of patients with bilateral ureteral stoma after radical cystectomy, a retrospective, noncomparative was performed. 544 patients with bilateral ureteral stoma after radical cystectomy in our hospital from December 2018 to December 2020 were selected. The self-designed questionnaire, discharge readiness scale (RHDS) and discharge guidance quality scale (QDTS) were used to investigate the general data, and multiple linear regression was used to analyze the related influencing factors. The total score of RHDS was (72.57 ± 18.56) and the total score of QDTS was (105.63 ± 24.18); the total score of RHDS was positively correlated with the total score of QDTS (*r* = 0.882, *p* = 0.000); the results of multiple linear regression showed that age, discharge direction and care mode were the main factors influencing the discharge readiness of patients (*p* < 0.05). In conclusions, the discharge readiness of patients with bilateral ureteral stoma after radical cystectomy is in the medium level, and there is a large space for improvement. Nurses should strengthen the guidance and nursing of patients’ discharge preparation to reduce the incidence of postoperative complications and readmission rate.

## Introduction

Bladder cancer is a common malignancy of the urinary system, which occurs mostly in elderly men, and the incidence is increasing year by year (1). According, novel diagnosis tool has been developed to make better diagnosis and decision. For example, growing evidence has shown that liquid biopsy biomarkers, such as assays of DNA methylation and mutations, protein-based assays, specific mRNA, and even non-coding RNAs, are of great value for the diagnosis and choice of the urinary diversion (2). Also, novel imaging technologies, such as Vesical Imaging-Reporting and Data System, has been developed and exhibit excellent advantage in detection of muscle invasive bladder cancer and thus influence the clinical choice for surgery (3). To data, radical cystectomy following bilateral ureteral abdominal wall stoma has considered to be one of the main treatment options for patients with bladder cancer (4). These patients need to wear an ostomy bag for the rest of life, which not only changed the patient’s self-image, but also cause a serious burden on the patient’s psychology, physiology, and economy after the operation (5). For instance, in addition to changes in the urination method, the patient’s unfamiliar and difficult ways to replace the ostomy bag will cause great psychological pressure on the patient.

Readiness for discharge mainly refers to the ability of medical staff to provide patients with a comprehensive psychological, physical, and social assessment, and to further recover after the patient leaves the hospital and returns to the family and society (6). At this stage, the patient still faces a long recovery time after being discharged from the hospital and returning to the family and society. Many factors have been reported to affect readiness for hospital discharge in patients. For example, Qian et al. reported that total score of the QDTS, residence and educational level were the major influencing factors for readiness for hospital discharge in patients discharged with tubes (7). Similarly, another study showed that high education level, employment and the content-received dimension of QDTS favored participants’ readiness for hospital discharge (8).

Notably, another survey found that patients’ readiness for hospital discharge was lower in larger units and medical units but was greater in those with higher nurses’ experience, better patient self-reported health, higher patient ratings of self-care teaching and symptom management teaching in patients undergoing surgery (9). However, factors that affect discharge readiness of patients with bilateral ureteral stoma after radical cystectomy remain largely unknown.

This present study sought to explore the status of patients’ readiness for discharge and analyze relevant influencing factors. Our study will help to clarify the current problems and thereby grasp the requirements for treatment and rehabilitation after discharge.

## Methods

### Study Design and Clinical Information

A retrospective analysis was performed on all subjects undergoing bilateral ureteral abdominal wall stoma treatment after radical bladder cancer in the Second Xiangya Hospital between December 2018 to December 2020. A total of 544 patients were included. There were 338 males and 208 females with an average age of 76.3 years and an average hospital stay for 12.3 days. Inclusion criteria: All patients were diagnosed by surgical pathological examination and met the European Association of Urology (EAU) guidelines for the diagnosis and treatment of bladder cancer (10); all patients received bilateral ureteral abdominal wall stoma treatment after radical bladder cancer; age ≥18 years old; Patients without serious comorbidities; have basic communication and writing skills. Exclusion criteria: patients with cognitive dysfunction, mental or intellectual problems; patients with organic disease; patients with severe postoperative complications; patients with missing basic data and collected data.

### General Information Survey Questionnaire

Including patient gender, age, education, way of care, work status, marital status, preoperative complications, hospitalization time, urostomy complications, self-care ability score, presence, or absence of distant metastasis, urostomy complications.

### Readiness for Hospital Discharge Scale (RHDS) (11)

As assessed by the Chinese version of the RHDS scale, Cronbach’s α coefficient is 0.89, and the overall content validity index is 0.88, mainly including anticipatory support (4 items), adaptability (5 items), personal status (3 items) 3 dimensions, a total of 12 items, the validity index of each item ranges between 0.8–1.0 (11). The scale is evaluated by a 10-point assignment method. The total score of the scale is the sum of each item, with a score of 0–120. The higher the score, the better the patient’s readiness for discharge.

### Quality of Discharge Teaching Scale (QDTS) (12)

The quality of discharge guidance is assessed by the Chinese version of the QDTS scale (12). The Cronbach’s α coefficient of the scale is 0.924. The scale has three dimensions, including guidance skills and effects (12 items), the actual content (6 items), the content needed before discharge (6 items), a total of 24 items. The content validity index is 0.98, and the coefficient of each item is in the range of 0.882–0.935. Among them, the content needed before discharge corresponds to the content actually obtained, and the difference in scores between the two can be compared to judge the quality of discharge guidance. The QDTS scale is evaluated from 0–10 points. The total score are the sum of the actual content and guidance skills and effect scores. The higher the total score, the higher the quality of discharge guidance.

### Specific Surveys Method

Questionnaires were issued before the patients were discharged. The meaning of the questionnaires and related filling requirements were explained in detail during the questionnaire distribution. In this study, 544 questionnaires of each type were distributed, and 544 were recovered respectively, with a recovery rate of 100%.

### Statistical Analysis

The SPSS 19.0 statistical software was used to process the analysis. Continuous values were presented as means ± standard deviation (SD), while discrete values were showed using proportions. Mauchly’s Test following by Sphericity Assumed or modified Greenhouse-Geisser analysis was conducted to evaluate the Within-Subjects Effects over time and Between-Subjects Effects. Multilevel multivariate regression analysis was conducted to verify the relationships between the variables of patient’s age, whereabouts of discharge, education level, urostomy complications, occupation, hospital days and the way of care with the variable of patient readiness for hospital discharge. Difference at the level of *p* < 0.05 were considered statistically significant.

## Results

### Analysis of RHDS Scores and QDTS Scores in Patients with Bilateral Ureteral Abdominal Wall Stoma After Radical Operation of Bladder Cancer

The total score of RHDS was (72.57 ± 18.56) and the total score of QDTS was (105.63 ± 24.18). The detailed scores of each dimension of RHDS and QDTS are shown in Tables 1 and 2.

**Table 1:** RHDS scores of patients with bilateral ureteral abdominal wall stoma after radical bladder cancer surgery.

Dimensions	Total score	Actual score	Standardized score
Expected help	40	23.81 ± 6.58	1.96 ± 1.64
Coping ability	50	30.77 ± 7.84	2.53 ± 1.57
Personal status	30	17.88 ± 4.72	1.47 ± 1.58
Total score of the RHDS	120	72.57 ± 18.56	6.07 ± 1.53

**Table 2:** QDTS scores of patients with bilateral ureteral abdominal wall stoma after radical bladder cancer surgery.

Dimensions	Total score	Actual score	Standardized score
Teaching skills and effect	120	72.41 ± 16.88	6.08 ± 1.44
Actual obtained information	60	33.20 ± 7.88	5.52 ± 1.39
Required information	60	42.48 ± 3.74	7.05 ± 0.64
Total score of the QDTS	180	105.63 ± 24.18	5.87 ± 1.39

### Analysis of RHDS Scores in Patients with Bilateral Ureteral Abdominal Wall Stoma After Radical Resection of Bladder Cancer with Different Characteristics

Significant differences in the discharge readiness scores of patients with bilateral ureteral abdominal wall stoma after radical bladder cancer surgery with different education levels, ages, care styles, work status, discharge destination, length of stay, urostomy complications, and home-to-hospital distance (*p* < 0.05). See Table 3 and Supplementary Table S1 for details.

**Table 3:** Analysis of RHDS scores in patients with different characteristics (*n *= 544).

Items		Score of the RHDS	Statistics	*p* value
Age			*F* = 21.554	<0.001
18 ≤ age ≤ 44	32	90.53 ± 4.07		
45 ≤ age ≤ 74	160	83.11 ± 9.54		
>74	352	60.84 ± 17.61		
Gender			*t* = 0.311	0.735
Male	336	73.52 ± 19.67		
Female	208	71.23 ± 16.90		
Main caregiver after discharge			*F* = 12.781	< 0.001
Self	88	85.49 ± 9.76		
Spouse	288	78.37 ± 13.28		
Children	152	64.79 ± 17.84		
Others	16	41.09 ± 14.48		
Education level			*F* = 4.511	0.007
Junior high school or less	100	73.62 ± 15.52		
High school	284	82.77 ± 13.23		
College or higher	160	90.07 ± 3.51		
Urostomy complications			*t* = 7.875	<0.001
Yes	212	59.49 ± 19.16		
No	332	80.24 ± 13.47		

### Correlation Analysis of Patient Discharge Guidance Quality and Discharge Readiness

In patients with bilateral ureteral abdominal wall stoma after radical bladder cancer, RHDS is positively correlated with QDTS guidance skills, effects, and content obtained, and the total scores of the two are positively correlated (r = 0.882, *p* = 0.000), see Table 4 for details.

**Table 4:** Correlation analysis between patient discharge guidance quality and discharge readiness (*n *= 544, *r* value).

Items	Expected help	Coping ability	Personal status	Total score of the RHDS
Total score of the QDTS	0.908	0.863	0.837	0.884*
Teaching skills and effect	0.842	0.819	0.781	0.833
Actual obtained information	0.961	0.896	0.877	0.911*
Required information	0.181	0.114	0.001	0.092

* *p < 0.05.*

### Multivariate Analysis of Influencing Factors of Patients’ Readiness for Discharge from Hospital

The statistically significant variables in the single factor were used as independent variables, and the dependent variable was the total score of RHDS, and multiple linear regression analysis was performed. The results showed that the patient’s age, whereabouts of discharge, and the way of care were the main factors affecting the readiness for discharge of patients with bilateral ureterostomy after radical bladder cancer surgery (*p* < 0.05), see Table 5.

**Table 5:** Multivariate analysis of influencing factors of patients’ discharge readiness.

Variable	Regression coefficients	Standardized regression coefficient	*t* value	*p* value
Constant	132.473	–	20.255	0.000
Age	−12.387	−0.434	−2.967	<0.001
Discharge location	−10.588	−0.265	−3.774	<0.001
Main caregiver after discharge	−7.24	−0.353	−4.865	<0.001

## Discussion

The total RHDS score of patients with bilateral ureterostomy after radical resection of bladder cancer was (72.54 ± 18.37), which was at a medium level, and there was a large room for improvement. It may be related to the characteristics of the patient’s disease. Patients need to wear an ostomy bag for life after surgery, and the physiological structure changes cause great psychological pressure on patients. In addition, most patients have insufficient knowledge of the disease, surgery and stoma, and the long-term rehabilitation effect is poor, which will have a negative impact on the patient’s discharge readiness (13). Further analysis was made on the scores of all dimensions of patients’ discharge readiness, among which the score of personal state dimension after standardized score was the lowest (1.47 ± 1.53). The analysis may be attributed to the fact that under the concept of quick health, the length of hospital stay of patients was significantly shortened, and some patients met the discharge indicators but did not fully recover. Discharge at this time would increase the pressure of discharge of patients. Therefore, nursing staff should do a good job in the discharge evaluation of planned discharged patients, especially those with poor discharge readiness, to further reduce the readmission rate of patients and improve the discharge readiness of patients (14, 15).

QDTS are widely used to evaluate whether patients perceive the teaching ability of nursing and thereby verify the discharge education skills of the nurses, which was demonstrated to have high reliability with modification to Chinese version (16, 17). Herein, we adopted QDTS to evaluate the level of pre-discharge education. Interestingly, QDTS was positively associated with RHDS, indicating the important role of nursing skills and pre-discharge education in enhancing readiness of discharge.

Multiple linear regression analysis showed that discharge destination was the main factor influencing the discharge readiness of patients with bilateral ureterostomy after radical resection of bladder cancer (*p* < 0.05). Returning home directly after discharge indicates that patients have a good recovery condition and have family members or partners to take care of them, which can significantly improve patients’ sense of security and recovery confidence (8). However, patients transferred to rehabilitation institutions predict more complications, poor prognosis, relatively complex follow-up treatment for patients, prone to anxiety, tension, and other adverse emotions, reducing the patient’s discharge readiness. For such patients, palliative care services can be provided. Patients can first transition to rehabilitation centers or community hospitals and other institutions to improve the quality of care, and then realize the smooth transition from institutions to families, reducing the incidence of complications on the basis of reducing medical costs (9).

The results of multiple linear regression analysis showed that the way of care was the main factor affecting the readiness for discharge of patients with bilateral ureterostomy after radical bladder cancer surgery (*p* < 0.05). Patients living alone are relatively poorly prepared for discharge. Analysis of the reasons shows that because bladder cancer is high in the elderly, most of the spouses are retired, and they can provide life care and companionship for patients, and emergency situations can be dealt with and help in time, and patients have a relatively higher sense of safety when discharged from the hospital (18). Patients living alone have poor self-care ability, cannot fully grasp the method of ostomy bag replacement and stoma care methods in the short term, encounter emergencies or difficulties in life, cannot get help and support from family members, and the patient’s sense of security is relatively low. Some patients cared for by their children, due to their fast-paced life and busy work, are unable to provide patients with long-term companionship, and most patients are prone to guilt and are unwilling to trouble their children, leading to relatively poor readiness for discharge from the hospital (19).

Multiple linear regression analysis showed that age was the main factor influencing the discharge readiness of patients with bilateral ureterostomy after radical resection of bladder cancer (*p* < 0.05). With the increase of age, the body function of patients gradually decreases, hearing and vision are significantly reduced, more basic diseases are prone to occur, and the self-care ability of patients is relatively poor (20). Moreover, most elderly patients have relatively low acceptance of health knowledge and are difficult to master knowledge and skills such as pocket replacement operation and self-ostomy nursing in a short period of time, and do not recognize the self-care ability after discharge. In addition, older patients tend not to actively express their own needs and concerns, resulting in nursing staff unable to carry out specific discharge guidance according to the actual needs of patients, patients’ needs cannot be met, resulting in patients’ lack of coping skills and confidence for possible complications in the future, and relatively high discharge pressure. Nursing staff can ensure the continuity and coordination of care through effective nursing plans such as discharge education, health follow-up and drug guidance, and improve the patient’s discharge readiness (21).

However, certain limitations should be addressed, including single center team setting. Moreover, our analysis is based on retrospective data that could not be well-controlled.

Anyhow, our present study provides data for understanding the readiness status of patients with bilateral ureteral abdominal wall stoma after radical bladder cancer.

In summary, the readiness for discharge of patients with bilateral ureteral abdominal wall stoma after radical bladder cancer surgery is at a moderate level, and there is a lot of room for improvement. Nursing staff should strengthen the guidance and care of patients’ discharge preparations to reduce the incidence of postoperative complications and the rate of readmission.

## Data Availability

The original contributions presented in the study are included in the article/Supplementary Material, further inquiries can be directed to the corresponding author/s.
