# Supplementary material for: Status Quo and Influencing Factors of Discharge Readiness of Patients with Bilateral Ureteral Stoma After Radical Cystectomy
Source: Front Surg. 2022 May 25;9:860162. doi: 10.3389/fsurg.2022.860162 (PMC9407034; doi:10.3389/fsurg.2022.860162)
Supplement: Supplementary file 1 [file Table_1_v1.docx]

**Table 1**

**Supplementary Table 1 Analysis of RHDS scores in patients with different characteristics（n=544）**

| Items |  | Score of the RHDS | Statistics | P value |
| --- | --- | --- | --- | --- |
| **Occupation** |  |  | F=4.738 | 0.013 |
| Employment | 200 | 82.66±13.79 |  |  |
| Retired | 304 | 68.30±18.61 |  |  |
| Others | 40 | 94.46±9.33 |  |  |
| **Marital status** |  |  | F=1.533 | 0.226 |
| Single | 216 | 90.61±3.77 |  |  |
| Married | 288 | 71.83±18.59 |  |  |
| Others | 40 | 69.71±18.46 |  |  |
| **Distance from home to hospital** |  |  | F=5.664 | 0.002 |
| ＜1.0 km | 136 | 73.84±18.39 |  |  |
| 1.0-4.9 km | 216 | 79.63±11.49 |  |  |
| 5.0-10.0 km | 152 | 62.66±19.83 |  |  |
| ＞10.0 km | 40 | 51.74±21.72 |  |  |
| **Discharge location** |  |  | t=4.344 | ＜0.001 |
| Transfer to rehabilitation facility | 168 | 58.19±20.33 |  |  |
| Home | 376 | 79.25±13.48 |  |  |
| **Activities of daily living** |  |  | F=1.458 | 0.151 |
| ≤40 scores | 28 | 41.38±23.27 |  |  |
| 41≤scores≤60 | 132 | 74.16±14.88 |  |  |
| 61≤scores≤100 | 384 | 74.11±17.83 |  |  |
| **Hospitalized days** |  |  | t=2.258 | 0.028 |
| ＜10 days | 360 | 85.83±13.27 |  |  |
| ≥10 days | 184 | 70.51±18.60 |  |  |

**Table 4 Correlation analysis between patient discharge guidance quality and discharge readiness (n=544, r value)**

| Items | Expected help | Coping ability | Personal status | Total score of the RHDS |
| --- | --- | --- | --- | --- |
| Total score of the QDTS | 0.908 | 0.863 | 0.837 | 0.884 |
| Teaching skills and effect | 0.842 | 0.819 | 0.781 | 0.833 |
| Actual obtained information | 0.961 | 0.896 | 0.877 | 0.911 |
| Required information | 0.181 | 0.114 | 0.001 | 0.092 |

**Table 5 Multivariate analysis of influencing factors of patients' discharge readiness**

| Variable | Regression coefficients | Standardized regression coefficient | t value | P value |
| --- | --- | --- | --- | --- |
| Constant | 132.473 | - | 20.255 | 0.000 |
| Age | -12.387 | -0.434 | -2.967 | ＜0.001 |
| Discharge location | -10.588 | -0.265 | -3.774 | ＜0.001 |
| Main caregiver after discharge | -7.24 | -0.353 | -4.865 | ＜0.001 |
